# Supplementary material for: Characterization and Preliminary In Vitro Antioxidant Activity of a New Multidrug Formulation Based on the Co-Encapsulation of Rutin and the α-Acylamino-β-Lactone NAAA Inhibitor URB894 within PLGA Nanoparticles
Source: Antioxidants (Basel). 2023 Jan 28;12(2):305. doi: 10.3390/antiox12020305 (PMC9951992; doi:10.3390/antiox12020305)
Supplement: Supplementary file 1 [file antioxidants-12-00305-s001.zip › antioxidants-2149452-supplementary.pdf]

Characterization and Preliminary *In Vitro* Antioxidant Activity of a New Multidrug Formulation Based on the Co-Encapsulation of Rutin and the α-Acylamino-β-Lactone NAAA Inhibitor URB894 within PLGA Nanoparticles

Agnese Gagliardi[a,§], Silvia Voci[a,§], Nicola Ambrosio[a], Massimo Fresta[a], Andrea Duranti[b,*] Donato Cosco[a,*]

[a]Department of Health Sciences, University “Magna Græcia” of Catanzaro, Campus Universitario “S Venuta”, 88100, Catanzaro, Italy;

[b]Department of Biomolecular Sciences, University of Urbino Carlo Bo, Piazza del Rinascimento 6, 61029, Urbino, Italy

Supplementary material

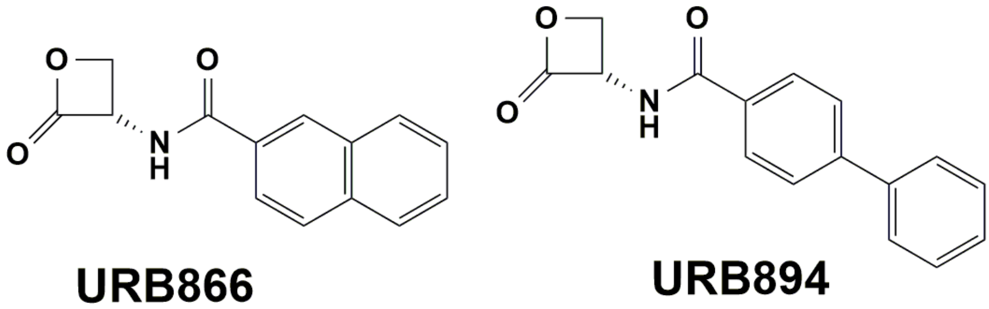

Fig. S1. Chemical structures of URB866 and URB894.

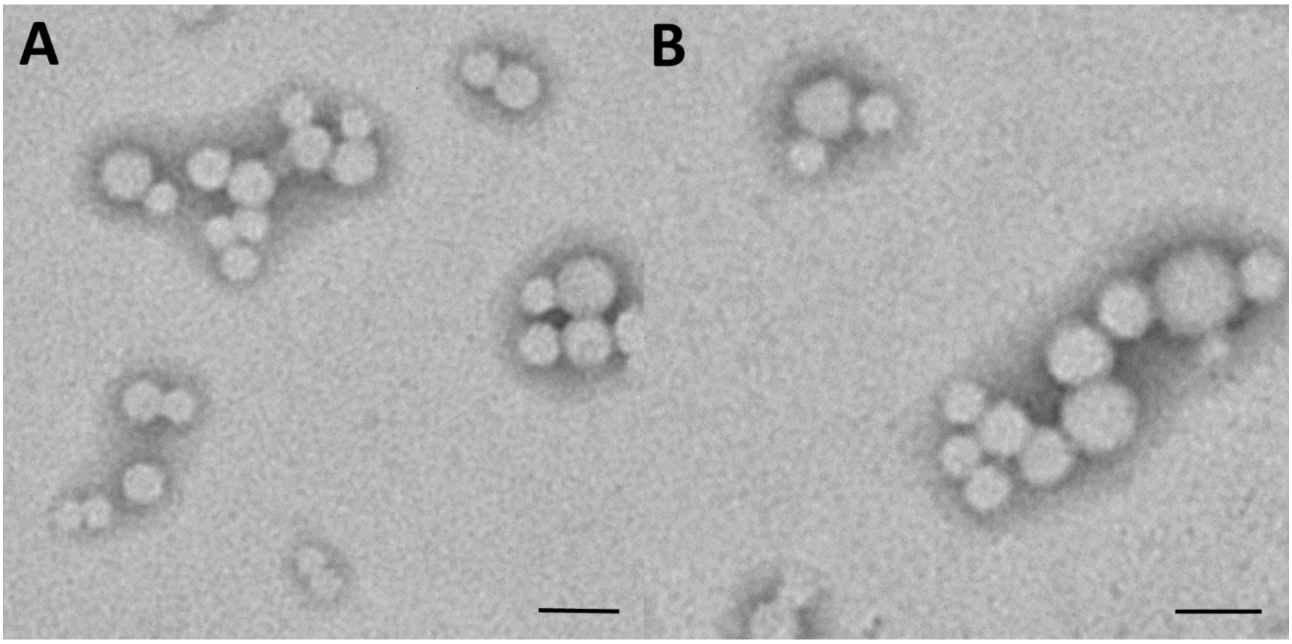

Fig. S2. TEM images of **(A)** empty nanosystems and **(B)** PLGA-based nanoparticles prepared with 0.4 mg/mL of URB894 and 0.6 mg/mL of rutin. Scale bar = 200 nm.
